# FOXK1 Promotes Proliferation and Metastasis of Gallbladder Cancer by Activating AKT/mTOR Signaling Pathway

**DOI:** 10.3389/fonc.2020.00545

**Published:** 2020-04-17

**Authors:** Ma Wencong, Wang Jinghan, Yu Yong, Ao Jianyang, Li Bin, Cheng Qingbao, Liu Chen, Jiang Xiaoqing

**Affiliations:** Department of Biliary Tract Surgery I, Third Affiliated Hospital of Second Military Medical University, Shanghai, China

**Keywords:** gallbladder cancer, prognosis, FOXK1, progression, AKT/mTOR

## Abstract

Gallbladder cancer (GBC) is one of the most lethal malignancies worldwide, with extremely poor prognosis. Recently, forkhead box k1 (FOXK1), a member of the FOX transcription factor family, has been reported to be correlated with tumor progression in multiple malignancies. However, the role of FOXK1 in GBC has not been elucidated. In this study, we demonstrated that the expression level of FOXK1 was elevated in human GBC tissues and associated with increased liver metastasis, poor histological differentiation, advanced TNM stage, and shorter overall survival. Knockdown of FOXK1 expression inhibited GBC cells proliferation and metastasis. Consistently, overexpression of FOXK1 promoted GBC cells progression. Mechanical investigations verified that knockdown of FOXK1 could lead to G1/S cell cycle arrest through downregulating CDK4, CDK6, cyclin D1, and cyclin E1. And FOXK1 could regulate the expression of epithelial–mesenchymal transition (EMT) related proteins E-cad, N-cad, and Vimentin. Moreover, we found that FOXK1 could regulate the activation of Akt/mTOR signaling pathway. In addition, AKT special inhibitor MK-2206 could abolish the proliferation and metastasis discrepancy between FOXK1 overexpression GBC cells and control cells, which suggested the tumorpromoting effect of FOXK1 may be partially related with the activations of Akt/mTOR signaling pathway. Collectively, our results suggested that FOXK1 promotes GBC cells progression and represent a novel prognostic biomarker and potential therapeutic target in GBC.

## Introduction

Gallbladder cancer (GBC) is an aggressive and most common malignancy of the biliary tract system, accounting for 80–95% of biliary cancers (1). Currently, the most efficient therapeutic method for GBC is still the complete surgical resection (2). However, owing to its non-specific symptoms, early local infiltration and distant metastasis, most patients are diagnosed at advanced stage and lost their best surgery chance. Moreover, many GBCs are discovered incidentally at the time of laparoscopic cholecystectomy, and only 10–25% of these tumors could be completely resected (3). The prognosis of GBC is extremely poor with a 5-year survival rate of 5–10% (1, 4). Therefore, a better understanding of the molecular mechanisms of GBC is indispensable for discovering novel and effective therapeutic approaches for the treatment and identifying new biomarkers that can reflect therapeutic responses.

The forkhead box (Fox) gene family, which is characterized by the evolutionary conserved “forkhead” or “winged-helix” DNA-binding domain, encode a group of highly conserved transcription regulators (5–7). Through the transcriptional control of gene expression, FOX protein members have been demonstrated to be involved in embryonic development (8), organogenesis (9), and also multiple physiological processes including cell signaling (10), metabolic processes (11, 12), cell cycle control (13), immune responses (14), and even cognitive function (15). Meanwhile, dysregulation of FOX proteins has been reported to promote the development and progress of disorders, especially carcinogenesis (16, 17). Forkhead box k1 (FOXK1), a member of the FOX transcription factor family, has been reported to play pivotal roles in cell cycle control (18), cell signaling (19), and cell metabolism (20). Recently, accumulating evidences showed that FOXK1 was upregulated in various tumor tissues and might participate in the tumorigenesis (19, 21). However, the expression pattern and effect of FOXK1 on the development, progression and prognosis of GBC has never been investigated.

In the present study, we found that the expression of FOXK1 was upregulated in GBC tissues and correlated with the poor prognosis of GBC patients. Biological function study demonstrated that FOXK1 overexpression promoted the proliferation and metastasis of GBC cells. Further mechanism study reveals that FOXK1 interference could downregulate phosphorylation of AKT and mTOR in GBC cells.

## Materials and Methods

### Patients and Specimens

Formalin-fixed paraffin-embedded (FFPE) cancer tissue specimens used for immunohistochemistry analysis were obtained from 97 GBC patients who underwent radical cholecystectomy without preoperative chemotherapy, or radiotherapy between Feb 2013 and Feb 2016 at the Department of Biliary Tract Surgery I, Third Affiliated Hospital of Second Military Medical University. The detailed clinical and pathological information of these 97 GBC patients was summarized in Table 1. In addition, 64 FFPE gallbladder tissue samples achieved from patients with cholelithiasis, who underwent laparoscopic cholecystectomy, were included as controls. Fresh GBC tissue samples and matched adjacent non-tumor tissue samples were collected from an independent set of 42 GBC patients from March 2017 to April 2018 and stored at −80°C within 15 min after removal. These samples were used for quantitative real-time PCR analysis (qRT-PCR). All FFPE specimens and fresh tissue samples were confirmed by two pathologists and the clinical and pathological diagnosis was determined according to 8th AJCC criteria. The present study was approved by the Ethics Committee of our hospital and each enrolled patient signed a written informed consent before sample collection.

**Table 1:** Correlation of FOXK1 expression with the clinicopathological characteristics of GBC.

	**Cases**	**FOXK1 expression**		**χ^**2**^-value**	***P*-value**
		**Low**	**High**		
Sex				0.011	0.918
Male	33	11	22		
Female	64	22	42		
Age (years)				0.311	0.577
<60	42	13	29		
≥60	55	20	35		
Gallbladder stone				0.505	0.477
Present	66	24	42		
Absent	31	9	22		
Histology differentiation				4.279	**0.039**
Well/moderate	73	29	44		
Poor	24	4	20		
Lymph node metastasis				1.731	0.188
Present	32	8	24		
Absent	65	25	40		
Liver metastasis				6.820	**0.009**
Present	64	16	48		
Absent	33	17	16		
TNM stage (AJCC)				6.220	**0.013**
0-II	26	14	12		
III-IV	71	19	52		

*p-values represent Pearson χ^2^-test*.

*Bold values indicate statistical significance, P < 0.05*.

### Cell Lines and Cell Culture

The GBC cell line GBC-SD was obtained from the Chinese Academy of Life Sciences (Shanghai, China). The cell line NOZ, SGC-996, and EH-GB1 was kindly supplied by Xinhua hospital (Shanghai, China).

NOZ was maintained in William's medium E (Gibco, USA) and SGC-996 was cultured in RPMI 1640 (Hyclone, USA). EH-GB1 and GBC-SD was grown in high-glucose DMEM (Hyclone, USA). All the cells were grown in the medium supplemented with 10% fetal bovine serum (Gibco, USA) and 1% antibiotics in a 5% enriched CO_2_ atmosphere at 37°C.

### Vector Construction and Lentivirus-Mediated RNA Interference

The full-length FOXK1 cDNA (GenBank accession number NM_001037165) was amplified and subcloned to into pCDNA3.1 expressing vector (Invitrogen, Carlsbad, CA). The expression level of FOXK1 was then examined by western blot. Empty vector- transfected cells were used as a control.

Small interfering RNA (siRNA) targeting FOXK1 and negative control was specifically synthesized by GenePharma (Shanghai, China). Lipofectamine RNAiMAX (Thermo Fisher Scientific, Waltham, MA) was used to transfect siRNAs into cells according to the manufacturer's protocol. The siRNA sequences were as follows: siFOXK1 sense strand, 5′-GCAUGGGCCUGUCCAGCUUT−3′; the scrambled (src) siRNA 5′-TTCTCCGAACGTGTCACGT-3. The siRNA-transfected cells were harvested in the medium for 2 days after transfection and then used for subsequent studies.

Lentiviral vectors containing human FOXK1 short-hairpin RNA (Lv-shFOXK1) and scrambled non-targeting shRNA (Lv-shNC) which was served as negative control were provided by Genechem Company (Shanghai, China). The guiding strand sequences of Lv-shRNA were as follows: shFOXK1, 5′- CCTCTCTCTTAACCGCTACTT-3′; shNC, 5′- TTCTCCGAACGTGTCACGT-3′; Cells were infected with indicated lentivirus at an multiplicity of infection (MOI) of 20 for 48 h and then selected with puromycin (1.5 μg/mL) for 7 days. The expression level of FOXK1 in the infected cells was validated by western blot analysis.

### Immunohistochemical Analysis and Evaluation of FOXK1 Expression

Immunohistochemical staining was conducted on human samples and xenograft tumors following a standard staining procedure as previously described (22). FOXK1 expression of the tissue specimens was evaluated in a blinded manner by two pathologists without prior knowledge of the study design. The scores of the staining were determined on the intensity and percentage of the immunoreactive cells. Staining intensity was scored as 0 (negative), 1 (weak), 2 (moderate), and 3 (strong). And the staining range was graded as 0 (0% stained), 1 (1–25% stained), 2 (26–50% stained), and 3 (51–100% stained). The final score defined as the sum of two parameters was classified into four groups as negative (0), weak (1–2), moderate (3), and strong (4–6) staining. For statistical analysis, the moderate and strong group was defined as positive, and the others were defined as negative.

### RNA Extraction and Quantitative Real-Time PCR

Total RNA was isolated from GBC tissues or cell lines with Trizol reagent (Invitrogen, CA). The cDNA was synthesized by using reverse transcriptase M-MLV (Takara, Japan) according to the manufacturer's instructions. To detect the expression level of target genes, real-time PCR was performed on the ABI Prism 7500 system (Applied Biosystems, CA) with the SYBR Premix Ex Taq (Takara, Japan) using the 2^−ΔΔCT^ method. Data were normalized by internal control β-actin. The primers for FOXK1 were as follows: forward: 5′-TCCAGGAGCCGCACTTCTA-3′;

reverse: 5′- CTCCGGGATGTGGATCTTCA-3′; The primers for β-actin were as follows: forward: 5′- CGTGGACATCCGTAAAGACC−3′; reverse: 5′-ACATCTGCTGGAAGGTGGAC -3′.

### Western Blot Analysis

The proteins of tissues and cells were lysed in M-PER Protein Extraction Reagent (Pierce) mixed with protease inhibitor cocktail. BCA assay kit (Pierce) was used to determine the protein concentrations. The proteins were then separated on 8% SDS-PAGE, and transferred onto nitrocellulose membranes. After blocked with 5% non-fat milk for 1.5 h at room temperature, the membrane were incubated with primary antibodies overnight at 4°C, and followed by incubation with the corresponding secondary antibodies for 1.5 h. The blots were detected by ECL chemiluminescence kit (Millipore). These primary antibodies were obtained from Cell Signaling Technology (Danvers, USA), FOXK1 (# 12025), mTOR (# 2983), p-mTOR (# 5536), AKT (# 4691), p-AKT (# 4060), Cyclin D1(# 2922), Cyclin E1 (# 4129), E-cadherin (# 3195), N- cadherin (# 13116), CDK4 (# 12790), CDK6 (# 3136), and anti-Rabbit IgG-HRP (# 7074). The antibody against vimentin (#ab8979) was purchased from Abcam (Cambridge, USA). The antibody against Gapdh (# 60004-1-lg) and anti-Mouse IgG-HRP (# SA00001-1) were purchased from Proteintech (Rosemont, USA). Selected blots were quantified by using Image J (NIH, USA).

### Cell Proliferation Assay

A Cell Counting Kit-8 cell proliferation assay (CCK-8; Dojindo, Japan) was conducted according to the manufacturer's instructions. Briefly, 1000 GBC cells in 100 μL medium were seeded into each well of 96-well-plates. At the indicated time point (1, 2, 3, 4, 5 d), 10 μL CCK8 was added to the each well of the plates for 2 h at 37°C. Then, we measured the absorbance of the plates at 450 nm.

### Plate Colony Formation Assay

For the plate colony formation assay, 600 cells were separately seeded into 6-well-plates with 2.5 ml medium and then maintained at 37°C with 5% CO_2_. After culturing for 14 days, the cells were fixed with 4% paraformaldehyde for 15 min and stained with 0.1% crystal violet for 30 min. The number of clones (>50 cells/colony) was counted and all the assays were repeated three times.

### Cell Apoptosis and Cell Cycle

Cell apoptosis and cell cycle were analyzed through flow cytometry. Cell apoptosis was measured using the Annexin V-APC/7-AAD apoptosis kit according to the manufacturer's recommendation (BioLegend, USA). Briefly, GBC-SD, NOZ and SGC-996 cells were seeded in 6-well-plates (5 × 10^5^ cells/well) and transfected with FOXK1 siRNA or negative control RNA. The cells were washed with cold PBS twice, and then dissolved in 100 μL of 1 × binding buffer containing 5 μL of Annexin V-APC and 5 μL of 7-AAD. After incubation for 15 min at room temperature in the dark, 200 μL of the binding buffer was added to suspension. The stained cells were analyzed by flow cytometry (BD Biosciences). As for cell cycle analysis, the cells were collected and fixed with cold 70% ethanol for 24 h at 4°C. After incubated with PI/RNase staining buffer (BD Pharmingen, USA) for 30 min, the stained cells was analyzed by flow cytometry.

### Wound Healing Assay

For the *in vitro* wound-healing assay, cells were seeded on six-well-plates and cultured overnight. Then a cell-free area of the culture medium was generated by scratching with a 200 μL pipette tip. Cell migration into the wound area was measured in serum-free medium and photographed under a microscope at 0 and 48 h. All of the experiments were performed in triplicates.

### *In vitro* Migration and Invasion Assays

To evaluate the ability of migration and invasion, transwell assay and invasion chamber assay were performed in triplicate. In brief, transwell chambers for the twenty-four–well-plates with 8 μm pore size polycarbonate membrane (Corning, NY, USA) were applied in this assay. For migration assay, 2 × 10^4^ cells with 200 μl serum-free medium were seeded into the upper chamber and the lower chamber was filled with 600 μl medium with 10% fetal bovine serum. After 24 h, cells were fixed in 4% paraformaldehyde and stained with 0.1% crystal violet. The number of penetrated cells were counted in six random fields of each chamber and the mean values were then calculated. For the invasion assay. 4 × 10^4^ cells were seeded into the upper chamber with Matrigel (BD) coated membrane for 48 h. Three independent experiments were performed.

### *In vivo* Xenograft Tumor Studies and Metastasis Assays

NOZ-shNC (1 × 10^6^), and NOZ-shFOXK1 cells (1 × 10^6^) suspended in 100 μl phosphate-buffered saline (PBS) were subcutaneously injected into the right/left shoulder of 4-week-old female BALB/c nude mice which were purchased from the Animal Center of the Second Military Medical University. Tumor length and width were measured weekly with vernier calipers in a blinded manner. Tumor volume was calculated according to the following formula: V (mm3) = 4π/3 × width^2^ (mm2) × length/2 (mm). All mice were sacrificed for weight measurement and IHC staining of xenograft tumors 28 days after the injection.

For lung metastatic model, a total of 1 × 10^6^ NOZ-shNC or NOZ-shFOXK1 cells were injected into the tail veins of nude mice. Mice were sacrificed at 1 month post injection. The lung tissues of each mouse were separated and subjected to H&E staining. And the lung metastatic foci were counted in a double-blind manner with the aid of a dissecting microscope. All animal experiments were approved by the Animal Care Committee of our hospital.

### Statistical Analysis

Statistical analyses were performed using SPSS 22.0 and GraphPad Prism 6 software. Data are expressed as mean ± standard deviation (SD). Comparisons among groups were carried out with Student's *t*-test. The correlation between FOXK1 expression and clinicopathologic data was analyzed using the Pearson χ2 test. Kaplan-Meier method and log-rank tests were used for survival analysis. Univariate and multivariate Cox proportional hazard regression models were used to analyze independent prognostic factors. *P* < 0.05 were considered statistically significant.

## Results

### FOXK1 Expression Was Significantly Upregulated in Human Gallbladder Cancer Tissues

**Figure 1:**
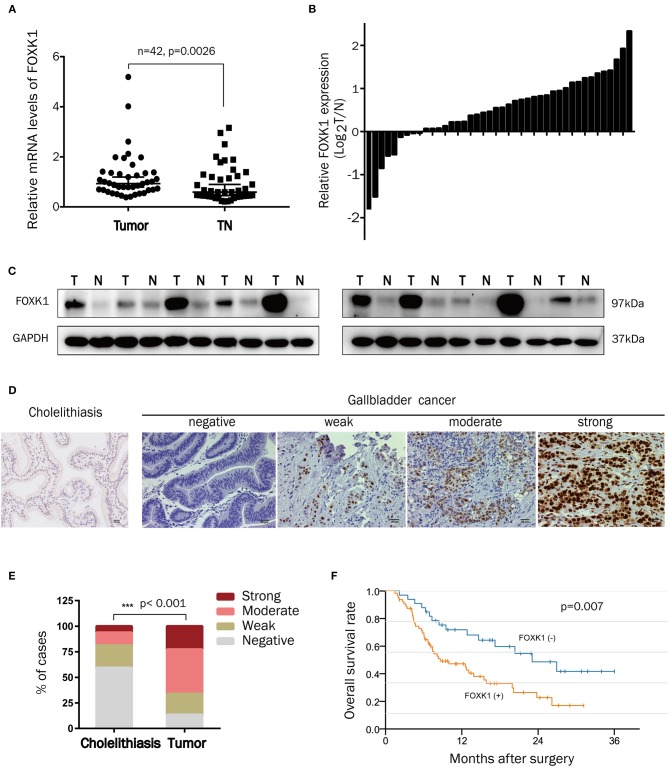
FOXK1 expression was upregulated in GBCs and correlated with overall survival. **(A,B)** Relative mRNA levels of FOXK1 in 42 paired human GBC tissues and adjacent normal tissues were determined by real-time qPCR. Gene expression results were normalized by GAPDH. **(C)** FOXK1 protein expression in 10 paired GBC tissues and their corresponding non-tumor tissues was determined by Western blot assay. **(D)** Representative images of IHC staining of FOXK1 in GBC tissues and non-tumor (cholecystitis) tissues (scale bar = 20 μm). **(E)** Percentage of cases with different staining intensity of FOXK1 in in the cholecystitis or GBC tissues. **(F)** Kaplan-Meier overall survival curve of GBC patients based on FOXK1 expression.

To assess the potential pathological role of FOXK1 in the development of GBC, the mRNA levels of FOXK1 in 42 pairs of GBC tumor and adjacent normal tissue samples were isolated and compared by qRT-PCR. As shown in Figures 1A,B, the relative mRNA level of FOXK1 was significantly higher in tumor tissues compared with that in their adjacent non-tumor tissues (*p* = 0.0026). Then, we examine the FOXK1 protein level by western blot assay and IHC staining assay. The western blot data showed that the protein level of FOXK1 was obviously increased in GBC tissues (*n* = 10) compared with matched adjacent normal tissue samples (Figure 1C). Immunohistochemistry analysis revealed that the positive staining of FOXK1 was mainly observed in the nucleus of cells and FOXK1 expression was significantly higher in tumor specimens compared with that in cholelithiasis tissues (Figures 1D,E). Among the 97 cases of GBC tissue samples, 23% (22/97) of cases were strongly stained, 43% (42/97) of cases were moderately stained, 21% (20/97) of cases were weakly stained and 13% (13/97) were negatively stained. On the contrary, only 6% (4/64) of the cholecystitis specimens showed strong staining of FOXK1 protein (Figure 1E).

### Increased FOXK1 Expression Correlates With Aggressive Clinicopathologic Features and Poor Prognosis in GBC Patients

In order to investigate the clinical significance of FOXK1 expression in GBC, we analyzed the correlation between FOXK1 expression and the clinicopathological characteristics in 97 GBC patients. As summarized in Table 1, high levels of FOXK1 expression were significantly correlated with increased liver metastasis (*p* = 0.009), histology differentiation (*p* = 0.039) and advanced TNM stage (*p* = 0.013). Furthermore, Kaplan-Meier survival analysis with the log-rank test revealed that patients in the high FOXK1 group had a significantly shorter overall survival (OS) than those in the low FOXK1 group (log rank, 11.301, *p* = 0.007, Figure 1F). To assess whether FOXK1 expression represents an independent prognostic factor in GBC patients, Cox univariate and multivariate regression analysis were conducted. In Cox univariate regression analysis, increased FOXK1 expression, poor histology differentiation, liver metastasis, advanced TNM stage, lymph node metastasis, and gallbladder stone were risk factors for OS. Moreover, when put these factors into Cox multivariate analysis, only lymph node metastasis, advanced TNM stage, gallbladder stone and increased FOXK1 expression were independent prognostic factors for OS in GBC patients (Table 2).

**Table 2:** Univariate and multivariate analysis of the association of prognosis with clinicopathological parameters and FOXK1 expression in GBC patients.

**Variable**	**Univariable analysis**		**Multivariable analysis**	
	**HR (95% CI)**	***P***	**HR (95% CI)**	***P***
Sex (male vs. female)	1.098 (0.637–1.893)	0.737		
Age (≥60 vs. <60)	1.054 (0.623–1.784)	0.844		
Gallbladder stone (present vs. absent)	1.860 (1.015–3.411)	**0.045**	2.551 (1.353–4.812)	**0.004**
Histology differentiation (Poor vs. Well/moderate)	1.782 (1.014–3.132)	**0.045**		
Lymph node metastasis (present vs. absent)	2.201 (1.274-3.803)	**0.005**	2.775 (1.529-5.037)	**0.001**
Liver metastasis (present vs. absent)	2.324 (1.249–4.326)	**0.008**		
TNM stage (III-IV vs. I-II)	2.621 (1.371–5.011)	**0.004**	2.585 (1.345–4.968)	**0.004**
FOXK1 expression (high vs. low)	2.236 (1.231–4.059)	**0.008**	1.896 (1.027-3.499)	**0.041**

*Bold values indicate statistical significance, P < 0.05, CI, confidence interval; HR, hazard ratio*.

### FOXK1 Promotes Proliferation Abilities of GBC Cells *in vitro*

**Figure 2:**
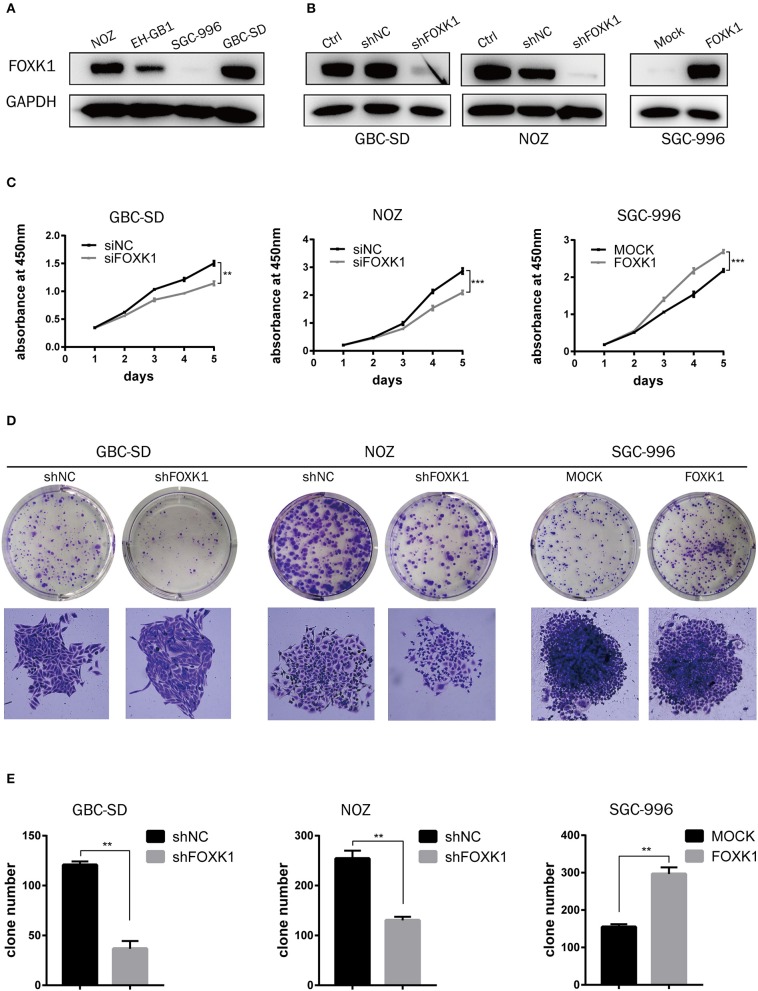
FOXK1 promotes GBC cell proliferation *in vitro*. **(A)** The protein levels of FOXK1 in four GBC cell lines. **(B)** Western blot analysis of FOXK1 expression in FOXK1-depleting GBC-SD and NOZ cells and FOXK1-overexpressingSGC-996 cells. GAPDH was used as the loading control. **(C)** The cell proliferation was determined by CCK-8 assays at various time points. **(D)** Effects of FOXK1 on the proliferation of GBC-SD, NOZ, and SGC-996 cells were assessed by the colony-forming assay. **(E)** The numbers of colonies were counted and depicted in the bar chart. Data shows the mean ± SD for three independent experiments. (*P < 0.05, **P < 0.01, and ***P < 0.001).

To explore the biological role of FOXK1 in the progression of GBC, we first performed western blot analysis to detect the endogenous expression of FOXK1 in GBC cell lines. Among the four GBC cell lines, the expression levels of FOXK1 in GBC-SD and NOZ cells were higher than that in EH-GB1 and SGC996 cells (Figure 2A). Then GBC-SD and NOZ cell lines were chosen for stable transfection with siRNA or shRNA lentivirus vectors toward FOXK1, and SGC-996 cell lines were chosen for stable transfection with FOXK1-expression vector. The efficiency of knockdown and overexpression of FOXK1 were verified through western blot (Figure 2B).

We perform CCK-8 and colony formation assays to investigate the effect of FOXK1 on the proliferation of GBC cells. As showed in Figure 2C, the proliferation of GBC-SD and NOZ cells was significantly suppressed by FOXK1 knockdown, while the cell proliferation was significantly enhanced by overexpression of FOXK1 in SGC996 cells (Figure 2C). Consistently, colony formation assay showed the same pattern. Knockdown of FOXK1 attenuated the colony formation capability of GBC-SD and NOZ cells, whereas overexpression of FOXK1 in SGC996 cells promoted colony formation compared with empty vector control cells (Figures 2D,E).

### Silencing of FOXK1 Leads to G1/S Cell Cycle Arrest

**Figure 3:**
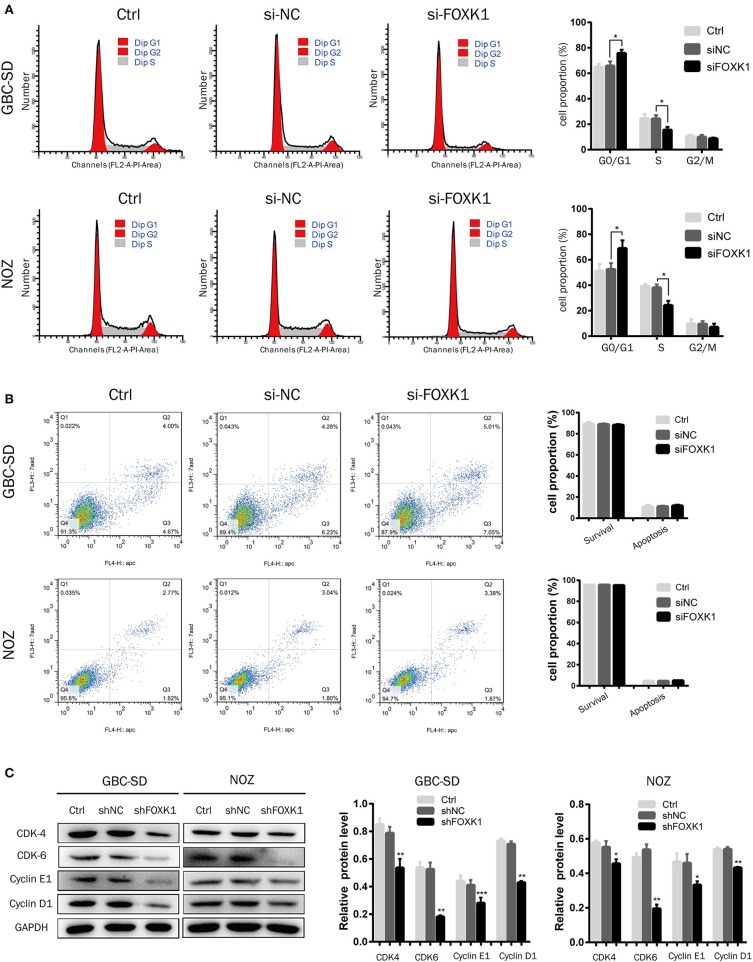
Silencing of FOXK1 leads to G1-S cell cycle arrest. **(A)** The cell cycle distribution of treated cells was analyzed by flow cytometry after transfection of siRNA for 48 h. Percentage of cells of different phase were shown in the bar chart (*P < 0.05, **P < 0.01, and ***P < 0.001). **(B)** The cells were stained with Annexin-V/7-AAD after transfection of siRNA for 48 h. The Q4 quadrant (Annexin V-/7-AAD-), Q3 quadrant (Annexin V+/7-AAD-), and Q2 quadrant (Annexin V+/7-AAD+) indicated the percentages of normal cells, cells in early apoptosis, and cells in late apoptosis, respectively. Percentage of surviving cells and apoptotic cells were depicted in the bar chart (*P < 0.05, **P < 0.01, and ***P < 0.001). **(C)** The cell cycle regulator CDK-4, CDK-6, cyclin E1, and cyclin D1 were detected by Western blot analysis with indicated antibodies, GAPDH was used as loading control. (*P < 0.05, **P < 0.01, and ***P < 0.001).

To study the mechanism by which FOXK1 promoted cell proliferation, we analyzed the effect of FOXK1 on apoptosis and cell cycle by flow cytometry analysis. The results demonstrated that knockdown of FOXK1 in NOZ and GBC-SD cells led to a significant increase in the G0/G1 phase cells, but a reduction in the S and G2/M phase population (Figure 3A). Then the cell cycle regulatory proteins associated with the G1/S phase transition was examined. As the western blot assay showed, the expression levels of CDK4, CDK6, cyclin D1, and cyclin E1were decreased when depletion of FOXK1 in NOZ and GBC-SD cells (Figure 3C). While we also found that FOXK1 had no significant effect on apoptosis of GBC cells (Figure 3B). These findings indicated that FOXK1 played an important role in the G1-S and S-G2/M phase transition which resulted in a rapid proliferation of GBC cells.

### Depletion of FOXK1 Inhibits Xenograft Tumor Growth

**Figure 4:**
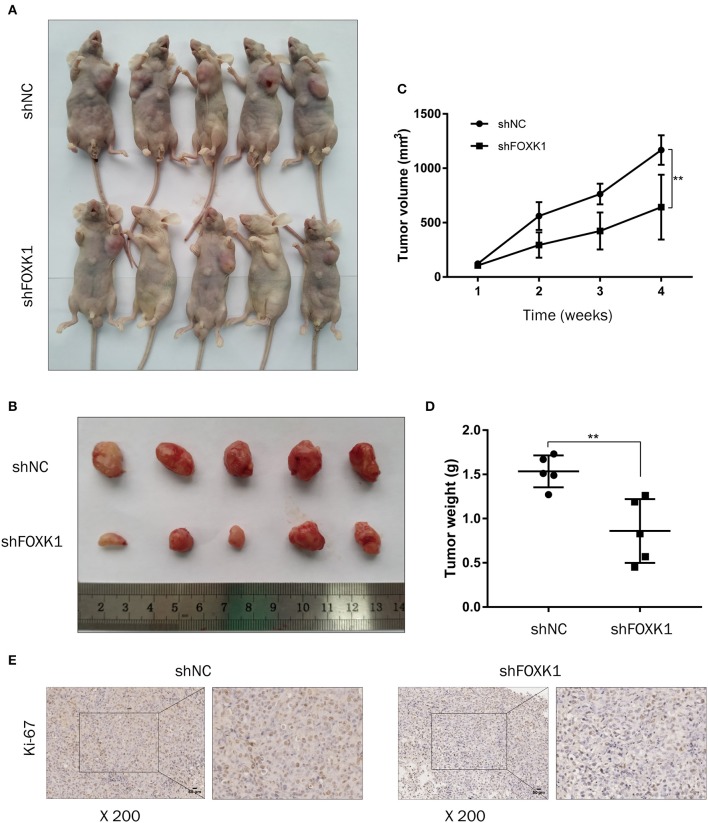
Depletion of FOXK1 attenuates xenograft tumor growth *in vivo*. **(A,B)** Representative images of sacrificed nude mice **(A)** and xenograft tumors **(B)** derived from subcutaneous implantation of NOZ cells treated with Lv-shFOXK1 or Lv-shNC. **(C)** Tumor growth curves in different group were calculated in a 4-week course (*P < 0.05, **P < 0.01, and ***P < 0.001). **(D)** Comparison of tumor weight between shFOXK1 group and shNC group (*P < 0.05, **P < 0.01, and ***P < 0.001). **(E)** Paraffin sections were stained for IHC with antibodies against ki-67, scale bar =20 μm.

To further evaluate the effect of FOXK1 on the tumor progression *in vivo*, xenograft tumor assay was performed in which FOXK1-depleted or control NOZ cells were injected into nude mice and tumor volume was monitored. The result showed that depletion of FOXK1 significantly inhibited xenograft tumor formation and growth (Figures 4A–D). Furthermore, IHC staining showed that cell proliferation marker Ki-67 was decreased in FOXK1-depleted tumor tissues (Figure 4E).

### FOXK1 Promotes GBC Cells Migration and Invasion *in vitro* and *in vivo* by Inducing EMT

**Figure 5:**
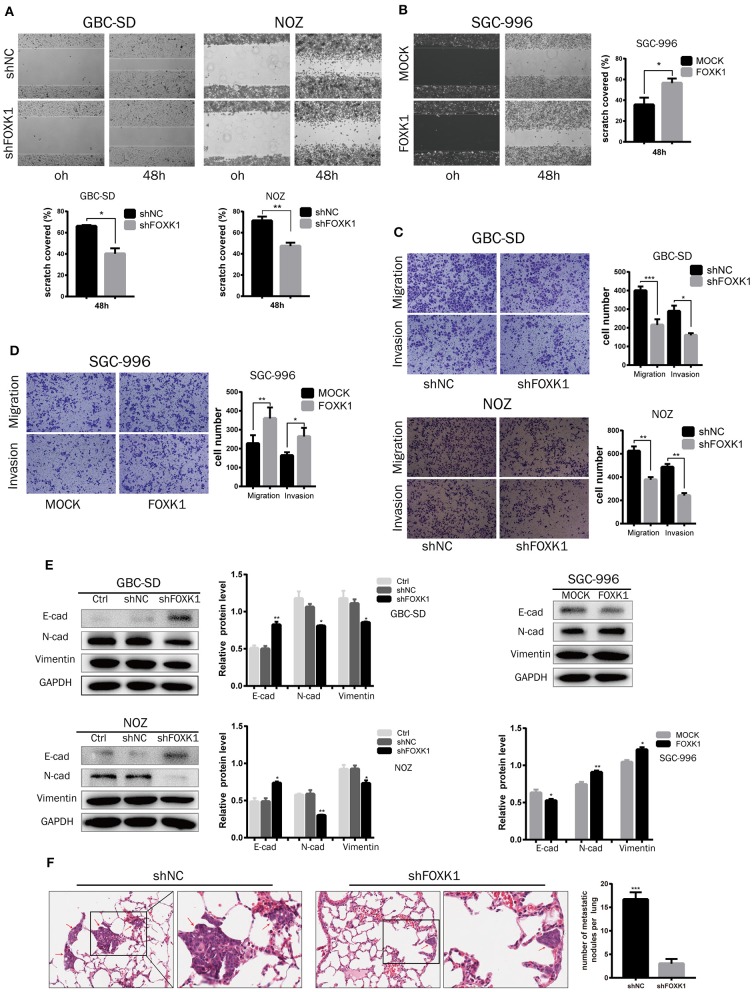
FOXK1 promotes tumor invasion and metastasis by inducing EMT. **(A,B)** For wound-healing assay, cell mobility was inhibited in FOXK1-depleting GBC-SD and NOZ cells. Overexpression of FOXK1 in SGC-996 cells had the opposite effects. Relative migration rate was shown in the bar chart. **(C,D)** Effects of FOXK1 on the migration and invasion of FOXK1-depleting GBC-SD and NOZ cells and FOXK1-overexpressingSGC-996 cells were determined by the transwell migration assay and matrigel invasion assay, respectively. The number of invaded cells were calculated and depicted in the bar chart. **(E)** The protein expression of N-cadherin, vimentin and E-cadherin in GBC cells was examined by Western blotting. **(F)** Representative images of H&E staining of lung metastases in each group. The number of metastatic nodules in the lung was calculated and compared in the bar chart. (*P < 0.05, **P < 0.01, and ***P < 0.001).

*In vitro* wound healing assay, transwell migration and invasion assay were conducted to investigate the role of FOXK1 in cell migration and invasion. The result showed that cell mobility and invasive capability was dramatically attenuated when knockdown of FOXK1 in NOZ and GBC-SD cells, while overexpression of FOXK1 in SGC-996 cells demonstrated the opposite effect (Figures 5A–D). As EMT is recognized as an important process in migration and invasion of cancer cells, we then detected the changes of EMT-associated biomarkers in these cells by western blot assay. We found that the expression levels of E-cadherin were enhanced while N-cadherin and Vimentin were reduced in both GBC-SD and NOZ cells transfected with shFOXK1. In contrast, overexpression of FOXK1 in SGC-996 cells could significantly reduce E-cadherin but increase N-cadherin and Vimentin expression (Figure 5E). These results indicated that FOXK1 promotes GBC cells migration and invasion via inducing EMT.

To confirm these findings *in vivo*, we further conducted a lung tumor metastasis model. NOZ control or FOXK1-silenced cells were injected into the nude mice through the lateral tail vein to induce lung metastasis. As shown in the Figure 5F, the micrometastatic lesions in the group injected with NOZ-shFOXK1 cells were fewer and smaller compared with the group injected with control cells. Collectively, these findings suggested that FOXK1 is essential for the invasive and metastatic potential of GBC cells both *in vitro* and *in vivo*.

### FOXK1 Exerts the Tumor-Promoting Functions by Activating the AKT/mTOR Signaling Pathway

**Figure 6:**
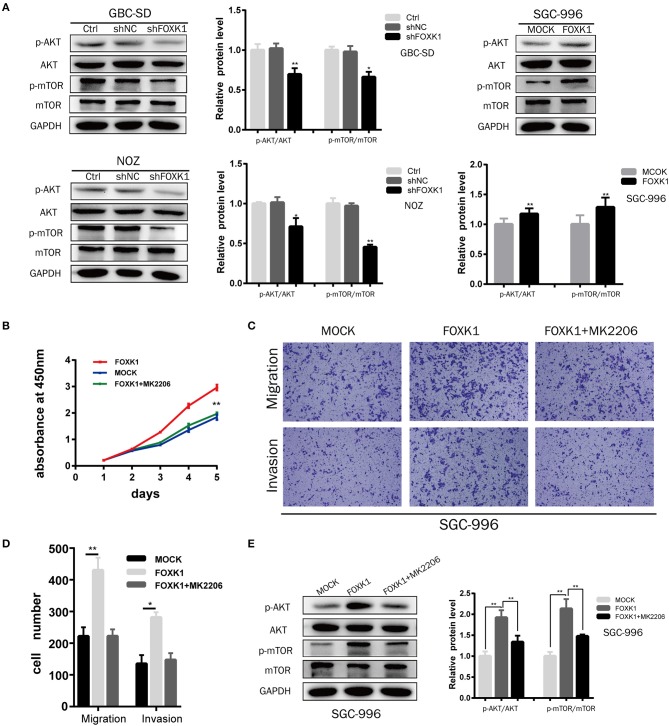
Akt/mTOR signaling pathway was involved in FOXK1-mediated proliferation, migration and invasion of GBC cells. **(A)** The protein expression of Akt, p-Akt(Ser473), mTOR, and p-mTOR (Ser 2448)in indicated cells was examined by Western blotting. **(B)** CCK-8 assay showed that the AKT special inhibitor MK2206 could abolish the enhanced proliferation by FOXK1 overexpression. **(C,D)** Transwell migration and matrigel invasion assay showed that the increasing mobility of GBC cells induced by FOXK1 overexpression was abrogated by MK2206. The number of invaded cells were calculated and depicted in the bar chart. **(E)** MK2206 reversed effects of FOXK1 overexpression on the expression of phosphorylated Akt (Ser473) and mTOR (Ser 2448) by Western blotting (*P < 0.05, **P < 0.01, and ***P < 0.001).

The AKT/mTOR signaling pathway has been demonstrated to be involved in the proliferation and metabolism of cells, thus playing an important role in the occurrence and development of cancers (23). Moreover, recent studies found that FOXK1 was enrolled in regulating metabolism (20). Therefore, we investigated whether FOXK1 exerts the tumor-promoting functions via Akt/mTOR signaling pathway. Compared with control cells, the expression levels of both phosphorylated Akt (Ser473) and mTOR (Ser 2448) were decreased in FOXK1 knockdown GBC cells (Figure 6A). Moreover, overexpression of FOXK1 in SGC-996 cells increased the phosphorylation of Akt (Ser473) and mTOR (Ser 2448) (Figure 6A). Then we treated FOXK1-overexpression GBC cell lines with the AKT special inhibitor MK2206 (25 μM), which inhibits the phosphorylation of all AKT isoforms. The cell proliferation results showed that MK2206 could diminish the differences in proliferation between FOXK1-overexpression and control cells (Figure 6B). Accordingly, the elevated ability to invade and migrate of GBC cells induced by FOXK1 overexpression was abrogated by MK2206 (Figures 6C,D). Consistent with these observations, MK2206 reversed effects of FOXK1overexpression on the protein phosphorylation status of Akt (Ser473) and mTOR (Ser 2448) (Figure 6E).

## Discussion

GBC is a highly lethal and aggressive tumor and stubbornly resistant to standard forms of cytotoxic therapy (24). Radical resection may be a potential curative approach, but the prognosis is still poor (4, 25). It seems that advances in conventional therapy will impact slightly on survival, which highlights the need to find reliable tumor markers for early detection and cellular targets for novel therapeutic approaches. FOXK1 is a transcription factor which belongs to the forkhead family (5). Currently, growing evidence has indicated FOXK1 is overexpressed in various cancer tissues and functions as a oncogene (19, 26, 27). In this study, we elaborated the critical role of FOXK1 in GBC and the underlying mechanism. We also demonstrated the prognostic value of FOXK1 in GBC patients.

Herein, we investigated the expression level and biological role of FOXK1 in GBC for the first time. we found that FOXK1 expression was significantly up-regulated in GBC tissues compared with the cholecystitis gallbladder epithelial tissues and gallbladder normal tissue adjacent tumor. FOXK1 overexpression was correlated with liver metastasis, poor histology differentiation, advanced TNM stage and a shorter OS of GBC patients. Cox multivariate analysis further revealed that FOXK1 was an independent factor for poor prognosis. These clinical data strongly indicated that FOXK1 contributes to the progression and metastasis of GBC, and might be an independent prognostic factor for patients with GBC.

In this study, we confirmed that inhibition of FOXK1 decreased the GBC cell proliferation and tumor growth by a series of *in vitro* and *in vivo* assays. Through the ChIP-seq analyses, Gavin D. Grant et al. have revealed that FOXK1 could bind to the promoters of an expansive set of genes which were critical for cell cycle progression (18). Therefore, we hypothesize that FOXK1 change the cell proliferation through cell cycle control. Further flow cytometry analyses proved the hypothesis and demonstrated the that knockdown of FOXK1 leads to G1/S cell cycle arrest, while with no obvious effect on apoptosis, which was consistent with previous research result (28).

Additionally, we also found that inhibition of FOXK1 attenuated the migrative and invasive ability of GBC cell lines *in vitro* and *in vivo*, indicating that FOXK1 might be involved in tumor metastasis, which happens very early in GBC patients. Metastasis is a process that cancer cells attenuate cell-cell adhesion and disseminate into distant organs, in which epithelial-mesenchymal transition (EMT) plays a critical role. EMT is an orchestrated series of events, in which epithelial cells lose their properties and acquire mesenchymal phenotypes, resulting in a loss of epithelial polarity and reduced intercellular adhesion (29). Recently, an increasing studies have reported that the Forkhead transcription factor family members including FOXK1 induce EMT and aggressiveness in human cancer (16, 17, 30). Consistently, our study revealed that slicing of FOXK1 increased the expression of epithelial marker E-cadherin and reducing the expression of mesenchymal marker vimentin and N-cadherin. While overexpression of FOXK1 showed the opposite effect. These findings indicated that FOXK1 might play a role in tumor metastasis.

Recently, studies have shown that FOXK1 was likely to act as an important regulator in cellular metabolism. Sukonina et al. found that the increased glucose uptake induced by overexpression of FOXK1 or FOXK2 is entirely used for aerobic glycolysis and production of lactate, which is an important intermediary in numerous metabolic processes (20). Another study showed that FOXK1 knockdown inhibit glycolysis in liver cancer (31). It is well-known that Akt/mTOR signaling pathway was frequently activated in cancer and controlled cell metabolism and growth (23, 32). Then, we put forward the hypothesis that the tumor-promoting effect of FOXK1 might be associated with the Akt/mTOR signaling pathway. Moreover, FOXK2, one of the two members of FOXK family, which collaborated with FOXK1 and showed the similar capability in a few physiological and pathological processes (19, 20, 33), has been demonstrated to change the phosphorylated status of AKT and exert oncogenic function in hepatocellular carcinoma (34). Therefore, we further evaluate if FOXK1 promote the tumorigenesis of GBC through activating the Akt/mTOR signaling pathway. Our results showed that slicing of FOXK1 significantly decreased the levels of both phosphorylated Akt (ser 473) and mTOR (ser 2448) in GBC-SD and NOZ cells, with no detectable changes in total protein levels of Akt and mTOR. On the contrary, overexpression of FOXK1 in SGC996 cells elevated the levels of p-Akt (ser 473) and p-mTOR (ser2448). Moreover, Akt special inhibitor MK-2206 could diminish the proliferation and metastasis discrepancy between FOXK1 overexpression GBC cells and control cells, and the increasing level of phosphorylated Akt and mTOR induced by overexpression of FOXK1 was reversed by MK2206. This indicated that, the increased mTOR phosphorylation upon FOXK1 overexpression might through the enhanced level of phosphorylated Akt, which could inhibit the phosphorylation of PRAS40 resulting in the elevating activity of mTOR. The underlying specific mechanism for FOXK1 to activate Akt signaling cascade might be correlated with its transcriptional regulation of gene expression and this need to be further investigated by more experiments, such as ChIP-seq. Collectively, the enhanced GBC malignant phenotype due to overexpression of FOXK1 could be explained, at least in part, through activating the Akt/mTOR signaling pathway. Of particular note is that recent study reported that mTORC1 inhibited the phosphorylation of FOXK1 and the hypophosphorylated FOXK1 promote mTORC1-mediated metabolic reprogramming (35). Thus, whether there is a positive feedback regulation process between the FOXK1 and mTOR needs further study.

In conclusion, our study investigated for the first time the clinical and biological significance of FOXK1 in GBCs. We have demonstrated that the expression level of FOXK1 was upregulated in GBC tissue samples and cell lines and was correlated with advanced clinicopathological parameters and poor prognosis in GBC patients. Overexpression of FOXK1 promoted the cell proliferation by cell cycle control and enhanced the migrative and invasive ability of GBC cells through inducing the EMT. Moreover, the tumorpromoting effect of FOXK1 may be partially related the activations of Akt/mTOR axis. Therefore, FOXK1 may serve as a potential therapeutic target and prognostic marker for patients with GBC.

## Data Availability Statement

All experimental data of this study are included in the article.

## Ethics Statement

The studies involving human participants were reviewed and approved by The Ethics Committee of Third Affiliated Hospital of Second Military Medical University. The patients/participants provided their written informed consent to participate in this study. The animal study was reviewed and approved by The Ethics Committee of Third Affiliated Hospital of Second Military Medical University.

## Author Contributions

MW, WJ, LC, and JX contributed conception and design of the study. CQ, LB, and YY performed the samples collection. MW and AJ conducted the statistical analysis and interpretation of data. MW and WJ performed the experiments, produced the main draft of the manuscript and made figures. All authors contributed to manuscript revision, read and approved the submitted version.

## Conflict of Interest

The authors declare that the research was conducted in the absence of any commercial or financial relationships that could be construed as a potential conflict of interest.
